# A Description of the Isothermal Ageing Creep Process in Polymethyl Methacrylate Using Fractional Differential Models

**DOI:** 10.3390/polym16192725

**Published:** 2024-09-26

**Authors:** Chuhong Wang, Xin Chen

**Affiliations:** Key Laboratory of Green Building and Intelligent Construction in Higher Educational Institutions of Hunan Province, Hunan City University, Yiyang 413000, China; chenxin@hncu.edu.cn

**Keywords:** fractional differential models, nonlinear viscoelasticity, physical ageing, creep, PMMA

## Abstract

Fractional differential viscoelastic models can describe complex material behaviours and fit experimental data well; however, the physical significance of model parameters is difficult to express. In this study, the fractional differential Maxwell, Kelvin, and Zener models were used to fit the short-term creep compliance curves of polymethyl methacrylate at different ageing times. The model fits were in good agreement with the experimental data. As the ageing time increased, the fractional differential Zener model showed a relative increase in the modulus parameter of the spring and a relative decrease in the modulus parameter reflecting the viscosity of the spring-pot, which indicated that physical ageing made the material more elastic. The relaxation time of the material increased, which indicated that the physical ageing reduced the free volume of the material, hindered the movement of molecules/segments, and increased the time required for the material to reach equilibrium. The fractional order of the model decreased, which reflected the phenomenon that physical ageing reduced the creep compliance of the material. Using the relaxation time as the time scale, the creep curves at different ageing times under the same stress level could be superimposed, naturally presenting the time–ageing time equivalence principle.

## 1. Introduction

Amorphous glassy polymers with low density, superior impact resistance, and other excellent mechanical properties have been widely utilised in engineering fields, such as the automobile, aeronautical, medical, and electronic industries. Mechanical properties are the key to the promotion and application of glassy polymers in more potential aspects. Up to now, great efforts have been devoted to constructing constitutive models to describe the whole deformation process, i.e., three-stage stress evolutions. An efficient temperature model was particularly developed to describe the large strain hardening thermo-mechanical responses of PC [1]. Liu et al. [2] also established a constitutive model to explore temperature-sensitive viscoelastic–viscoplastic deformations of polycarbonate. An elastic–viscoplastic constitutive model was employed to characterise uniaxial compressions of PMMA [3]. As is well known, the elastic modulus and relaxation time are essential parameters for describing the mechanical behaviours of polymers. It was found that the elastic modulus monotonically decreases with an increasing temperature [4]. However, the considerable above-mentioned works aimed at increasing the precision of the constitutive model with cumbersome parameters. There is still a lack of effective models with fewer parameters for describing the mechanical behaviours of amorphous glassy polymers.

Polymer physical ageing refers to the process in which, below the glass transition temperature or after cooling from the processing or moulding state, the internal structure of a polymer material spontaneously transitions towards an equilibrium state over time. This process can lead to changes in the mechanical and physical properties of materials, such as an increase in a modulus, a decrease in toughness, and an increase in brittleness. These changes may affect the service life and performance of the material. Understanding the physical ageing behaviour of a polymer is crucial for predicting and extending its service life in various applications. Existing traditional models such as the Maxwell, Kelvin, and standard linear solid models often fail to fully capture the mechanical behaviours of polymers over long time scales. They cannot accurately describe the nonlinear characteristics typically exhibited during the ageing of a polymer. They may not effectively describe the memory effect of a material on its history. They have limitations in predicting the long-term behaviours of polymers under different temperatures and loading rates, and they may not accurately predict the ageing behaviours of polymers under changing environmental conditions.

Fractional differential models offer a new approach to overcoming these limitations. By introducing fractional derivatives, these models can more accurately describe the viscoelastic characteristics and time-dependent behaviours of polymers, thereby providing a better understanding of the physical ageing process. Fractional operators have an excellent preponderance in describing complex phenomena, like power law attenuation [5], nanofluid flowing [6], anomalous diffusion [7], biomedical engineering [8,9], and signal processing [10,11]. Subsequently, for the purpose of revealing large strain behaviours, the concept of variable order was introduced to characterise complex nonlinear stress–strain responses [12,13,14]. As the variable order is born with the ability to reflect the changes in mechanical properties under constant deformation, a variable-order model was successfully employed to characterise strain-softening phenomena [15,16,17]. Fractional models capture the long-term memory and hereditary characteristics of materials based on their parameters, thereby providing a new perspective and tool for polymer ageing research. Several relevant studies were recently conducted. Xu [18] proposed a fractional viscoelastic model specifically to describe the physical ageing behaviours of polymers. Li [19] used fractional calculus methods to model the physical ageing of polymers and provided a new theoretical perspective. Zhang [20] established a model based on fractional calculus to simulate the time-dependent behaviours of polymers. Li [21] combined experiments and simulations to propose a fractional model that describes the physical ageing of polymers. Mao [22] studied the physical ageing of polymers using fractional-order dynamic mechanical analysis. Wang [23] proposed a fractional model to predict the physical ageing of polymers.

The time–ageing equivalence principle is a concept in material science used to describe the behaviours of materials under different ageing times, and it is used particularly for polymeric materials. This principle posits that, through the appropriate transformation of time or ageing time, the behaviour of a material under various ageing conditions can be mapped onto a master curve. This principle is very useful for predicting the long-term performance of materials and understanding their physical ageing processes.

The long-term stability of the mechanical properties of polymethyl methacrylate (PMMA), which is widely used in construction and industry, is crucial for its applications. This study aimed to describe the creep behaviour of PMMA under different ageing conditions using fractional differential models and explore in depth the relationship between model parameters and physical ageing phenomena.

## 2. Materials and Methods

### 2.1. Definition of Fractional Calculus

Among the various definitions of fractional calculus, the Riemann–Liouville fractional calculus operator is widely used for viscoelastic polymer constitutive models [24]. The order α integral of function f(t) is defined as follows:

(1) $$I^{\alpha }f(t)=\int_{t_{0}}^{t}\frac{{\left(t-\tau \right)}^{\alpha -1}}{\Gamma (\alpha )}f(\tau )d\tau$$

where 0<α≤1, Γ(z) is a gamma function, and Γ(1+z)=z!.

When Re(z)>0, $\Gamma (z)=\int_{0}^{-\infty }e^{-t}t^{z-1}dt$, and when Re(z)<1, $\Gamma (z)=\frac{\pi }{\Gamma (1-z)\cdot \sin(\pi z)}$.

If $\bar{f}(p)$ is the Laplace transform of f(t), then the Laplace transform in the fractional calculus of f(t) is shown as follows:

(2) $$L\left[D^{\alpha }f(t),p\right]=p^{\alpha }\bar{f}(p)$$

### 2.2. Spring-Pot Model

**Figure 1:**
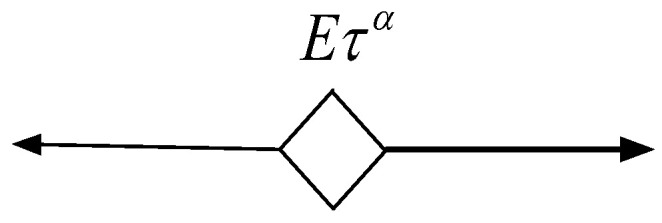
Spring-pot model.

Based on the fractional differential theory, Koeller [25] proposed a spring-pot element, as shown in Figure 1, to describe the viscoelastic behaviour of a material, with the constitutive relationship given by Equation (3):

(3) $$\sigma (t)=E^{1-\alpha }\eta^{\alpha }D^{\alpha }\varepsilon (t)=E\tau^{\alpha }D^{\alpha }\varepsilon (t)$$

In this equation, τ=η/E represents the average relaxation time of the spring-pot, and E represents the spring-pot’s elastic modulus. When α=1, the spring-pot degenerates into a Newtonian viscous fluid, and when α=0, the spring-pot degenerates into a spring. It can be seen that when 0<α<1, the spring-pot element characterises the viscoelastic behaviour of a material.

Performing a Laplace transform on Equation (3) with transform parameter p yields the following:

(4) $$\bar{\sigma }=E\tau^{\alpha }p^{\alpha }\bar{\varepsilon }$$

Under the action of step stress $\sigma_{0}H(t)$, where $\bar{\sigma }=\sigma_{0}/s$, Equation (4) can be transformed as follows:

(5) $$\bar{\varepsilon }(p)=\frac{\sigma_{0}}{E\tau^{\alpha }p^{\alpha +1}}$$

The Laplace inverse transform of the above gives the following:

(6) $$\varepsilon (t)=\frac{\sigma_{0}}{E\tau^{\alpha }}L^{-1}[p^{-\alpha -1}]$$

According to the properties of the Riemann–Liouville fractional calculus, $L^{-1}[p^{-\alpha -1}]=t^{\alpha }/\Gamma (1+\alpha )$, Equation (6) can be written as follows:

(7) $$\varepsilon (t)=\frac{\sigma_{0}}{E\Gamma (1+\alpha )}{\left(\frac{t}{\tau }\right)}^{\alpha }$$

Therefore, the creep compliance of the spring-pot is found as follows:

(8) $$J(t)=\frac{1}{E\Gamma (1+\alpha )}{\left(\frac{t}{\tau }\right)}^{\alpha }$$

Under the action of step strain $\varepsilon_{0}H(t)$, where $\bar{\varepsilon }=\varepsilon_{0}/p$, Equation (3) can be transformed into the following:

(9) $$\bar{\sigma }(p)=E\tau^{\alpha }p^{\alpha -1}\varepsilon_{0}$$

Performing the Laplace inverse transform yields the following:

(10) $$\sigma (t)=E\tau^{\alpha }\varepsilon_{0}L^{-1}\left[p^{\alpha -1}\right]=\frac{E\varepsilon_{0}}{\Gamma (1-\alpha )}{\left(\frac{t}{\tau }\right)}^{-\alpha }$$

Thus, the relaxation modulus of the spring-pot is found as follows:

(11) $$G(t)=\frac{E}{\Gamma (1-\alpha )}{\left(\frac{t}{\tau }\right)}^{-\alpha }$$

A single spring-pot cannot fully describe the complex viscoelastic behaviour of a material, and various fractional derivative models are generally composed of spring-pots and spring elements in series or parallel configurations.

### 2.3. Fractional Differential Maxwell Model

**Figure 2:**
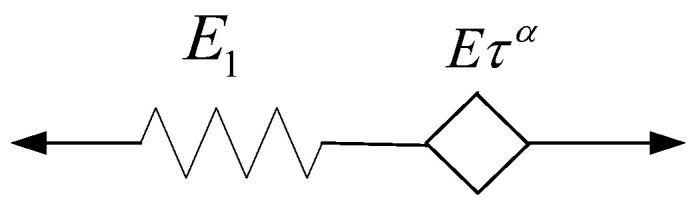
Fractional differential Maxwell model.

The fractional differential Maxwell model, which is composed of a spring-pot and spring in series, is shown in Figure 2. The stress–strain relationship of this model is given by Equation (12).

(12) $$\sigma (t)=\frac{E_{1}E\tau^{\alpha }D^{\alpha }}{E_{1}+E\tau^{\alpha }D^{\alpha }}\varepsilon \left(t\right)$$

where $E_{1}$ represents the elastic modulus of the series springs of all fractional differential model in this article.

Under the action of step stress $\sigma_{0}H(t)$, performing a Laplace transform and inverse transform on Equation (12) yields the following:

(13) $$\begin{array}{ll} \varepsilon \left(t\right) & =L^{-1}\left(\frac{E_{1}E\tau^{\alpha }p^{\alpha }}{E_{1}+E\tau^{\alpha }p^{\alpha }}\cdot \frac{1}{p}\right)\sigma_{0}=\frac{\sigma_{0}}{E_{1}}L^{-1}\left[\left(1+\frac{E_{1}}{E}{\left(\tau p\right)}^{-\alpha }\right)\frac{1}{p}\right]\\ \; & =\frac{\sigma_{0}}{E_{1}}L^{-1}\left[\frac{1}{p}+\frac{E_{1}}{E}\tau^{-\alpha }p^{-\alpha -1}\right]=\frac{\sigma_{0}}{E_{1}}\left[1+\frac{E_{1}}{E}{\left(\frac{t}{\tau }\right)}^{\alpha }\frac{1}{\Gamma \left(1+\alpha \right)}\right] \end{array}$$

Thus, its creep compliance is found as follows:

(14) $$J\left(t\right)=\frac{1}{E_{1}}\left[1+\frac{E_{1}}{E}{\left(\frac{t}{\tau }\right)}^{\alpha }\frac{1}{\Gamma \left(1+\alpha \right)}\right]$$

Similarly, under step strain $\varepsilon_{0}H(t)$, we have the following:

(15) $$\begin{array}{ll} \sigma \left(t\right) & =L^{-1}\left(\frac{E_{1}E\tau^{\alpha }p^{\alpha }}{E_{1}+E\tau^{\alpha }p^{\alpha }}\cdot \frac{1}{p}\right)\varepsilon_{0}=L^{-1}\left(\frac{E_{1}}{\frac{E_{1}}{E\tau^{\alpha }p^{\alpha }}+1}\cdot \frac{1}{p}\right)\varepsilon_{0}\\ \; & =L^{-1}\left[E_{1}\cdot \frac{1}{p}\cdot \sum_{k=0}^{\infty }{\left(-\frac{E_{1}}{E\tau^{\alpha }p^{\alpha }}\right)}^{k}\right]\varepsilon_{0}=L^{-1}\left[E_{1}\cdot \sum_{k=0}^{\infty }{\left(-\frac{E_{1}}{E\tau^{\alpha }}\right)}^{k}p^{-\alpha k-1}\right]\varepsilon_{0}\\ \; & =E_{1}\varepsilon_{0}\cdot \sum_{k=0}^{\infty }{\left(-\frac{E_{1}}{E\tau^{\alpha }}\right)}^{k}\frac{t^{\alpha k}}{\Gamma \left(\alpha k+1\right)}=E_{1}\varepsilon_{0}E_{\alpha ,1}\left[-\frac{E_{1}}{E}{\left(\frac{t}{\tau }\right)}^{\alpha }\right] \end{array}$$

In this equation, $E_{\alpha ,1}\left[-\frac{E_{1}}{E}{\left(\frac{t}{\tau }\right)}^{\alpha }\right]$ is the Mittag–Leffler function [26], which is defined as follows: $E_{\alpha ,1}\left(z\right)=\sum_{k=0}^{\infty }\frac{z^{k}}{\Gamma \left(\alpha k+1\right)}$.

Thus, its relaxation modulus is found as follows:

(16) $$G\left(t\right)=E_{1}E_{\alpha ,1}\left[-\frac{E_{1}}{E}{\left(\frac{t}{\tau }\right)}^{\alpha }\right]$$

The fractional differential Maxwell model has been widely applied to describe the creep behaviours of viscoelastic materials [27,28,29,30]. Chen [29] used the fractional differential Maxwell model to describe the long-term compressive creep behaviour of a type of salt rock and showed that the value of α was different in different creep stages. In the initial stage of creep, the salt rock was in a viscoelastic state, with α=0.37288; in the long-term creep stage, the salt rock was in a viscous flow state, with α=0.96467. Karner [30] used a fully fractional generalised Maxwell model to describe the time-dependent sinusoidal creep of a dielectric elastomer actuator and found that the implementation of the model in simulation control is straightforward. The proposed method can easily be used for other materials with viscoelastic behaviour.

### 2.4. Fractional Differential Kelvin Model

**Figure 3:**
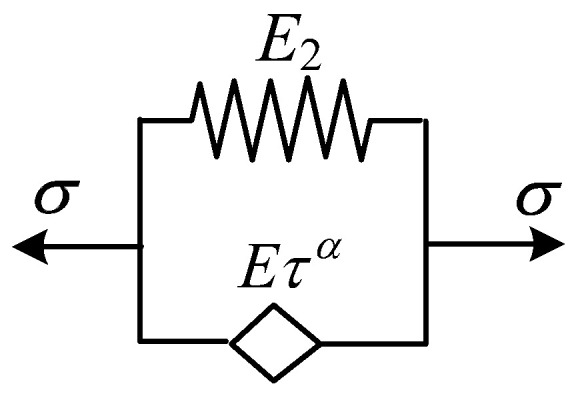
Fractional differential Kelvin model.

The fractional differential Kelvin model, which is composed of a spring and spring-pot in parallel, is shown in Figure 3. The stress–strain relationship is given by Equation (17).

(17) $$\sigma (t)=\left(E_{2}+E\tau^{\alpha }D^{\alpha }\right)\varepsilon (t)$$

where $E_{2}$ represents the elastic modulus of the parallel springs of all fractional differential models in this article.

Under the action of step stress $\sigma_{0}H(t)$, performing a Laplace transform and an inverse transform on Equation (17) yields the following:

(18) $$\begin{array}{ll} \varepsilon \left(t\right) & =L^{-1}\left(\frac{1}{E_{2}+E\tau^{\alpha }p^{\alpha }}\cdot \frac{1}{p}\right)\sigma_{0}=L^{-1}\left[\frac{p^{-\alpha -1}}{E_{2}p^{-\alpha }+E\tau^{\alpha }}\right]\sigma_{0}\\ \; & =\frac{\sigma_{0}}{E_{2}}E_{-\alpha ,1}\left(-\frac{E\tau^{\alpha }}{E_{2}t^{\alpha }}\right)=\frac{\sigma_{0}}{E}{\left(\frac{t}{\tau }\right)}^{\alpha }E_{\alpha ,1+\alpha }\left[-\frac{E_{2}}{E}{\left(\frac{t}{\tau }\right)}^{\alpha }\right] \end{array}$$

Thus, its creep compliance is as follows:

(19) $$J\left(t\right)=\frac{1}{E}{\left(\frac{t}{\tau }\right)}^{\alpha }E_{\alpha ,1+\alpha }\left[-\frac{E_{2}}{E}{\left(\frac{t}{\tau }\right)}^{\alpha }\right]$$

And its relaxation modulus is as follows:

(20) $$G\left(t\right)=E_{1}E_{\alpha ,1}\left[-\frac{E_{1}}{E}{\left(\frac{t}{\tau }\right)}^{\alpha }\right]$$

It can be seen that after the introduction of the spring-pot, the fractional differential Kelvin model can reflect the relaxation behaviour of a viscoelastic material through different values of α.

### 2.5. Fractional Differential Zener Model

**Figure 4:**
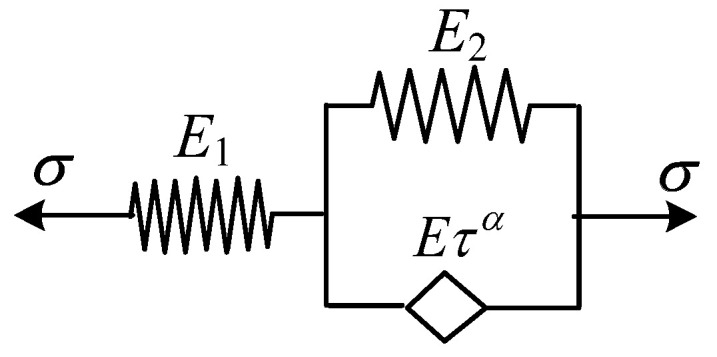
Fractional differential Zener model.

The fractional differential Zener model is obtained by replacing the viscous pot in the three-parameter solid model with a spring-pot. It can be constructed in two ways: either by connecting a spring and the fractional differential Kelvin model in series or by connecting a spring and the fractional differential Maxwell model in parallel. Taking the constitutive model shown in Figure 4 as an example, the stress–strain relationship is given by Equation (21):

(21) $$\sigma (t)=\frac{E_{1}\left(E_{2}+E\tau^{\alpha }D^{\alpha }\right)}{E_{1}+E_{2}+E\tau^{\alpha }D^{\alpha }}\varepsilon (t)$$

Similarly, under a step load, the creep compliance and relaxation modulus of the fractional differential Zener model can be obtained as follows:

(22) $$J\left(t\right)=L^{-1}\left[\frac{E_{1}+E_{2}+E\tau^{\alpha }p^{\alpha }}{E_{1}\left(E_{2}+E\tau^{\alpha }p^{\alpha }\right)}\cdot \frac{1}{p}\right]=\frac{1}{E_{1}}+\frac{1}{E_{2}}\cdot E_{-\alpha ,1}\left(-\frac{E}{E_{2}}{\left(\frac{t}{\tau }\right)}^{\alpha }\right)$$

(23) $$G\left(t\right)=L^{-1}\left[\frac{E_{1}\left(E_{2}+E\tau^{\alpha }p^{\alpha }\right)}{E_{1}+E_{2}+E\tau^{\alpha }p^{\alpha }}\cdot \frac{1}{p}\right]=E_{1}-\frac{E_{1}^{2}}{E_{1}+E_{2}}\cdot E_{-\alpha ,1}\left(-\frac{E}{E_{1}+E_{2}}{\left(\frac{t}{\tau }\right)}^{-\alpha }\right)$$

A comparison of the fractional differential Maxwell, Kelvin, and Zener models shows that the fractional differential Zener model is more comprehensive in describing the creep, relaxation, and dynamic mechanical behaviours of viscoelastic materials than the fractional differential Maxwell and Kelvin models.

### 2.6. Materials and Experiments

The experimental material was a commercial PMMA sheet with a thickness of 1.8–2.2 mm. The specimens were cut into dumbbell shapes using a laser cutter to meet the GB/T1040-2006 standards [31]. The experiments were conducted on a CSS44020 electronic tensile testing machine to ensure the consistency and repeatability of the experimental conditions.

The specimens were placed in a 202-OA-type electric thermal constant-temperature drying oven, heated to 115 °C, and maintained at that temperature for 30 min. The door of the constant-temperature drying oven was opened to allow the temperature to naturally decrease to 27 °C, and the temperature was kept constant at 27 °C to record the physical ageing time. Uniaxial tensile creep tests were conducted on the specimens with different ageing times at 15 and 25 MPa.

## 3. Results

**Figure 5:**
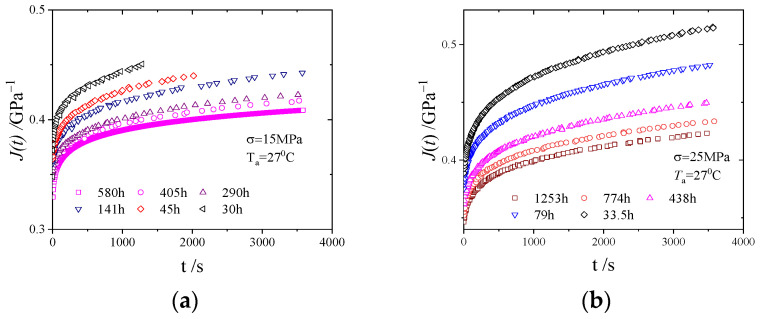
(**a**) Creep compliance of PMMA at 15 MPa. (**b**) Creep compliance of PMMA at 25 MPa.

The creep compliance curves are shown in Figure 5, from which it can be seen that the creep compliance of PMMA decreased with increasing physical ageing time.

Figure 5 shows the creep compliance curves of PMMA at different ageing times under stresses of 15 and 25 MPa. As the ageing time increased, the creep compliance decreased significantly, indicating that the mechanical properties of the material gradually degraded.

**Figure 6:**
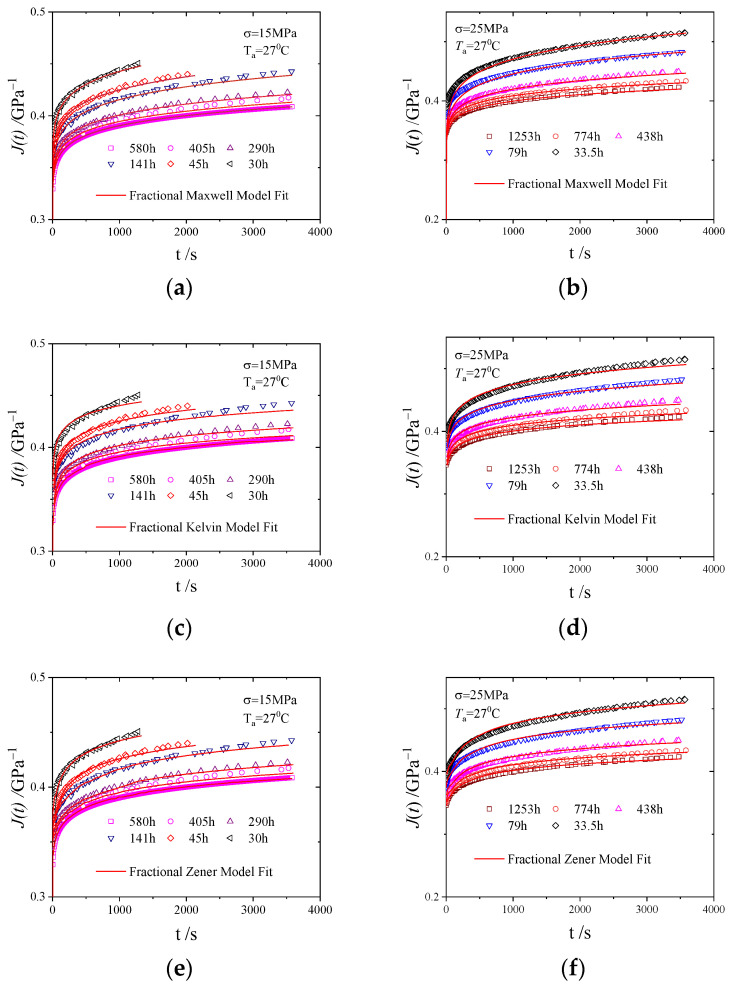
(**a**) Fractional differential Maxwell model at 15 MPa. (**b**) Fractional differential Maxwell model at 25 MPa. (**c**) Fractional differential Kelvin model at 15 MPa. (**d**) Fractional differential Kelvin model at 25 MPa. (**e**) Fractional differential Zener model at 15 MPa. (**f**) Fractional differential Zener model at 25 MPa.

The experimental data were fitted using Equations (14), (19), and (22), and the parameters were optimised using the Marquardt method (Levenberg–Marquardt) and a general global optimisation method with the smallest error of the experimental data fitting as the criterion. The fitting results are shown in Figure 6, and the obtained model parameters are listed in Table 1, Table 2 and Table 3. The root mean square error (RMSE) was controlled to within 0.1%, and the correlation coefficient (R) was close to one, indicating a good match between the model fitting and experimental data.

By analysing the parameter patterns in Table 1, Table 2 and Table 3, it can be seen that as the ageing time increased, the fractional differential Zener model showed a relative increase in the modulus parameter of the spring part and a relative decrease in the modulus parameter reflecting the viscosity of the spring-pot, indicating that physical ageing made the material more elastic. The relaxation time of the material increased, indicating that physical ageing reduced the free volume of the material, hindered the movement of molecules/segments, and increased the time required for the material to reach equilibrium. The fractional order of the model decreased, reflecting the phenomenon that physical ageing reduced the creep compliance of the material. Therefore, the fractional differential Zener model parameters listed in Table 3 are consistent with the actual physical significance of physical ageing.

**Figure 7:**
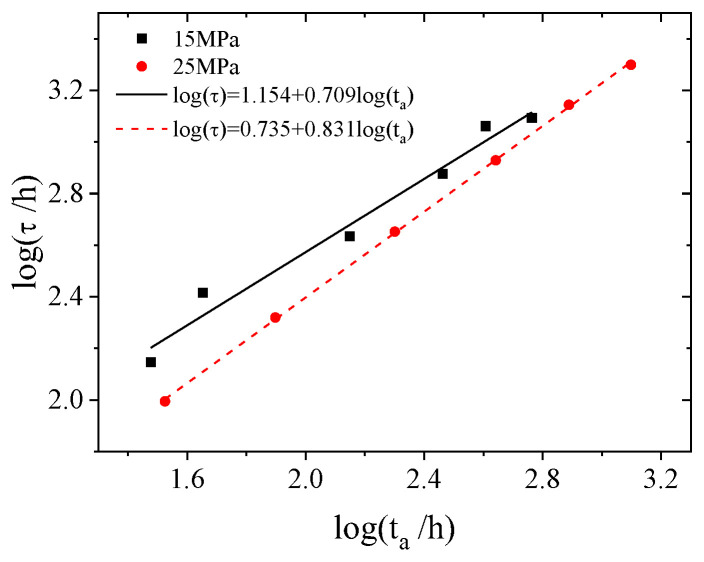
Relationships between relaxation time and ageing time at 15 MPa and 25 MPa.

Thermorheologically simple materials only have a glass transition, which is also known as the main transition. The time required for this transition is the relaxation time of the material, which has a power law relationship with the physical ageing time. Plotting the relaxation and physical ageing times from Table 3 in a double-logarithmic coordinate system, as shown in Figure 7, reveals a good linear relationship.

(24) $$\mathrm{At}\;15\;\mathrm{MPa}:\;\log\tau =1.154+0.709\log t_{a}$$

(25) $$\mathrm{At}\;25\;\mathrm{MPa}:\;\log\tau =0.735+0.831\log t_{a}$$

It can be seen that the slope of the fitting line at 25 MPa is 0.831, whereas at 15 MPa, the slope is 0.709, which indicates that under isothermal conditions and the same ageing time, increasing the stress level can accelerate the physical ageing of PMMA.

**Figure 8:**
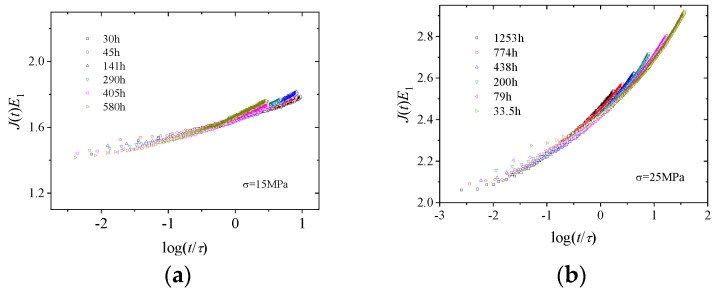
(**a**) $E_{1}J\left(t\right)$~t/τ curve at 15 MPa; (**b**) $E_{1}J\left(t\right)$~t/τ curve at 25 MPa.

The time–ageing time equivalence principle suggests that creep compliance curves at different ageing times can be superimposed onto a master curve after horizontal shifting by an ageing shift factor. If the relaxation time is used as the time scale and the creep compliance is normalised by multiplying it by a parameter, then the curves at different ageing times can also be superimposed onto a master curve by vertical shifting, as shown in Figure 8 for 15 and 25 MPa.

In summary, the fractional differential Zener model could accurately describe the physical ageing creep behaviour of PMMA. Using the relaxation time as the time scale, the creep curves at different ageing times under the same stress level could be superimposed, naturally presenting the time–ageing time equivalence principle.

## 4. Discussion

As seen from the fitting results shown in Figure 6, the model fitting at 25 MPa is better than that at 15 MPa; the reason for this is that the nonlinearity of high polymers is more significant under high stress [32]. Due to the strength limit of PMMA being about 30 MPa, the experimental stresses selected in this article are 15 MPa and 25 MPa, making them more comparable.

All the values of the fractional orders are observed to fall between 0 and 1 during complete deformation, which can intuitively reflect the mechanical properties. This is compatible with fractional viscoelastic theory, where the order set as 0 refers to pure elasticity, and 1 represents pure viscosity, as shown in Table 1, Table 2 and Table 3.

Considering the successful applications of fractional approaches in modelling complex mechanical responses and the corresponding finite element analysis [33,34,35], the proposed temperature-dependent fractional-order model will be extended to a three-dimensional one and deeply investigated with finite element analysis in the future to discuss the effects of more influential factors (such as temperature, variable load, etc.).

## 5. Conclusions

This study used the fractional differential Maxwell, Kelvin, and Zener models to analyse the physical ageing behaviour of PMMA and determine the physical significance of the parameters of these fractional differential models. The following briefly summarises the results.

(1) When fitting the creep data of PMMA at different ageing times with the fractional differential model, the Marquardt method (Levenberg–Marquardt) and a general global optimisation method were used to optimise the parameters, with the smallest error of the experimental data fitting as the criterion. The model fitting matched the experimental data well.
(2) As the ageing time increased, the fractional differential Zener model showed a relative increase in the modulus parameter of the spring and a relative decrease in the modulus parameter reflecting the viscosity of the spring-pot, which indicated that physical ageing made the material more elastic. The relaxation time of the material increased, which indicated that the physical ageing reduced the free volume of the material, hindered the movement of molecules/segments, and increased the time required for the material to reach equilibrium. The fractional order of the model decreased, which reflected the phenomenon that physical ageing reduced the creep compliance of the material.
(3) By plotting the relaxation time obtained from the fitting of the fractional differential Zener model and the physical ageing time in a double-logarithmic coordinate system, a linear relationship was obtained.
(4) Using the relaxation time as the time scale, the creep curves at different ageing times under the same stress level could be superimposed, naturally presenting the time–ageing time equivalence principle.

In summary, the fractional differential Zener model not only accurately described the physical ageing creep behaviour of PMMA but also provided an in-depth understanding of the changes in the internal structure of the material. Future research can further explore the application of this model to other polymer materials and optimise parameters to improve prediction accuracy. 

## Figures and Tables

**Table 1:** The parameters of the fractional differential Maxwell model.

T=27 °C	$t_{a}/h$	$E_{1}/\mathrm{GPa}$	E/GPa	τ/h	α	RMSE	R
15 MPa	30	4.383	7.31	24.972	0.441	0.0013	0.9968
	45	3.504	9.0	21.415	0.338	0.0041	0.9691
	141	4.105	6.67	37.65	0.438	0.0048	0.9619
	290	4.526	4.943	33.95	0.239	0.0051	0.9445
	405	2.684	5.687	36.574	0.434	0.0054	0.9317
	580	4.315	6.049	4.255	0.165	0.0061	0.9169
25 MPa	33.5	5.283	4.888	0.679	0.461	0.009	0.9724
	79	6.813	3.488	28.2	0.463	0.0091	0.9633
	438	5.924	4.203	834.26	0.459	0.0077	0.9376
	774	5.168	3.109	33.367	0.448	0.0073	0.9179
	1253	6.496	4.029	0.699	0.454	0.0075	0.9054

**Table 2:** The parameters of the fractional differential Kelvin model.

T=27 °C	$t_{a}/h$	$E_{2}/\mathrm{GPa}$	E/GPa	τ/h	α	RMSE	R
15 MPa	30	2.564	2.569	4.881	0.075	0.0105	0.9152
	45	2.801	2.939	3.139	0.103	0.018	0.926
	141	2.833	3.01	2.946	0.101	0.014	0.9944
	290	2.924	3.069	3.038	0.096	0.0135	0.9912
	405	2.921	3.044	3.144	0.087	0.0133	0.9411
	580	2.982	3.102	3.294	0.092	0.0071	0.9757
25 MPa	33.5	1.294	2.945	2.338	0.051	0.0348	0.9217
	79	7.662	10.439	9.574	0.011	0.0363	0.916
	438	0.003	3.039	2.761	0.039	0.0375	0.9829
	774	0.005	3.043	3.364	0.036	0.0397	0.9481
	1253	1.404	3.109	3.063	0.035	0.0389	0.9168

**Table 3:** The parameters of the fractional differential Zener model.

T=27 °C	$t_{a}/h$	$E_{1}/\mathrm{GPa}$	$E_{2}/\mathrm{GPa}$	E/GPa	τ/h	α	RMSE	R
15 MPa	30	2.774	2.131	36.89	139.872	0.1287	0.0018	0.961
	45	2.794	2.962	36.753	259.998	0.1248	0.0016	0.9023
	141	2.858	6.942	33.964	431.502	0.1008	0.0021	0.9546
	290	2.898	7.331	29.306	754.175	0.0826	0.0015	0.9424
	405	5.344	8.052	4.378	1152.72	0.0766	0.0021	0.9686
	580	6.232	8.574	2.991	1239.575	0.0677	0.0006	0.9581
25 MPa	33.5	2.591	4.847	200.39	98.698	0.1134	0.0032	0.9675
	79	2.669	5.879	137.5	208.704	0.0993	0.0026	0.9728
	438	2.771	6.921	33.49	848.867	0.0797	0.0022	0.9234
	774	2.839	7.708	28.88	1394.35	0.0673	0.0019	0.9934
	1253	2.896	8.277	7.494	1992.949	0.0635	0.0019	0.9671

## Data Availability

The original contributions presented in the study are included in the article, further inquiries can be directed to the corresponding author.
